# Can primary care data be used to monitor regional smoking prevalence? An analysis of The Health Improvement Network primary care data

**DOI:** 10.1186/1471-2458-11-773

**Published:** 2011-10-07

**Authors:** Tessa E Langley, Lisa C Szatkowski, Stephen Wythe, Sarah A Lewis

**Affiliations:** 1 Division of Epidemiology and Public Health, University of Nottingham, Nottingham, Clinical Sciences Building, Nottingham City Hospital, NG5 1PB, UK

## Abstract

**Background:**

Accurate and timely regional data on smoking trends allow tobacco control interventions to be targeted at the areas most in need and facilitate the evaluation of such interventions. Electronic primary care databases have the potential to provide a valuable source of such data due to their size, continuity and the availability of socio-demographic data. UK electronic primary care data on smoking prevalence from The Health Improvement Network (THIN) have previously been validated at the national level, but may be less representative at the regional level due to reduced sample sizes. We investigated whether this database provides valid regional data and whether it can be used to compare smoking prevalence in different UK regions.

**Methods:**

Annual estimates of smoking prevalence by government office region (GOR) from THIN were compared with estimates of smoking prevalence from the General Lifestyle Survey (GLF) from 2000 to 2008.

**Results:**

For all regions, THIN prevalence data were generally found to be highly comparable with GLF data from 2006 onwards.

**Conclusions:**

THIN primary care data could be used to monitor regional smoking prevalence and highlight regional differences in smoking in the UK.

## Background

Robust regional data on smoking prevalence are important for monitoring regional trends in smoking and evaluating the impact of national and regional tobacco control interventions. Currently, few large-scale high-quality regional data on smoking prevalence in the UK are available. The main source of regional prevalence data in the United Kingdom (UK) is national survey data. The General Lifestyle Survey (GLF), the current 'gold standard' for measuring smoking prevalence in Great Britain, has highlighted significant variation in smoking prevalence across the British regions [1]. However, survey data tend to be infrequently collected or have small sample sizes, particularly at the regional level, and there is often a significant time lag between the collection and the release of the data [2,3]. Electronic primary care databases have the potential to provide a valuable source of regional data on smoking prevalence due to their size, availability of monthly data and continuity.

The Health Improvement Network (THIN) (http://csdmruk.cegedim.com/) is a database of UK electronic primary care records. The validity of THIN data has been demonstrated for major events [4-9]. More recently, THIN data on smoking status have been validated at the national level. From 2006 onwards there was, despite the very different methods of obtaining prevalence estimates between the two data sources, generally good agreement between the prevalence of current smoking recorded in THIN and smoking rates based on national survey data from the GLF, suggesting that THIN may be an accurate means of monitoring smoking prevalence nationally [10]. However, the validity of THIN data on smoking prevalence at the regional level, which may be less precise as a result of smaller sample sizes and reduced representativeness, has yet to be demonstrated.

We therefore carried out a validation study comparing estimates of regional smoking prevalence from THIN with those from the GLF to assess whether THIN data can be used to monitor regional variation and trends in smoking prevalence.

## Methods

The version of THIN used in this study contains the primary care records of approximately 8 million patients from 446 general practices in England, Scotland, Wales and Northern Ireland, of whom 3.2 million are currently registered with a practice and can be followed prospectively; retrospective data is available for the remaining patients who have since either died or transferred from THIN practices. Prospective medical records are recorded using the Vision general practice computer system software, and serve as the primary medical record for the practice. GPs are able to record diagnoses, demographic information, lifestyle characteristics (such as smoking status) and other medical information. The dataset represents approximately 6% of the UK population [11].

Currently, the main source of statistics for monitoring smoking prevalence in Britain is a national, annual survey, the GLF [12], formerly known as the General Household Survey (GHS). It collects information on a range of topics from around 16,000 adults aged 16+ living in private households in England, Wales and Scotland each year. Topics include housing, employment, health, alcohol consumption and income as well as smoking, and data are available by age and sex. It uses a probability, stratified two-stage sample design and aims to interview all adults aged 16 or over at each sampled address [13]. In 2009 the response rate was 73% [1]. For this study, we used the GLF's measure of the prevalence of current cigarette smoking, which is obtained by asking respondents 'do you smoke cigarettes at all nowadays'. The data were weighted for non-response and also weighted to the population distribution of region, age group and sex.

The GLF covers England, Wales and Scotland only; therefore, the validation of the regional THIN prevalence data did not include data from Northern Ireland. The GLF surveys people aged 16 and over only and therefore under 16s were also excluded from this study. GLF data from 2000 to 2008, stratified by government office region (GOR), were used in this study.

For each year from 2000 to 2009 all live patients who were over the age of 16 and registered with a practice on an index date of 1st July of that year were identified from the THIN dataset and stratified by region. THIN data are regionally stratified by Strategic Health Authority (SHA). SHAs are coterminous with GORs, except that the South East region is divided into two: South Central and South East Coast. The THIN data for these two SHA regions were therefore combined. Patients who registered with a practice within the previous three months were excluded from this analysis (the GP contract requires that the smoking status of newly-registering patients is recorded within three months for this recording to be financially rewarded). The prevalence of smoking each year was calculated from the data recorded in medical records. All records of smoking status, identified by relevant Read Codes (a hierarchical dictionary of medical nomenclature [14]), entered into a patient's notes on or after their registration date were extracted. Patients were classified as current smokers at a given index date if their most recent smoking-related entry in their medical records prior to this index date identified them as such. The percentage of patients with no smoking status recorded decreased during the study period, from 36% in 2000 to 10% in 2009. A previous study has shown that the majority of patients with missing smoking records in THIN are ex or non-smokers, and therefore all patients with no smoking formation were assumed not to be current smokers at that point in time [15].

The way in which estimates of smoking prevalence are obtained are clearly very different in THIN and the GLF; however, the previous validation of THIN smoking prevalence data also used the GLF as a comparator and found that the estimates were comparable from 2006, suggesting that it is appropriate to compare these two data sources [10].

The sampling method of the GLF is designed to produce regionally representative estimates of smoking prevalence [13]. However, THIN comprises those GP practices in each region that have agreed to contribute their data to the database, and may not be regionally representative. We therefore initially compared the demographic structure of THIN at the regional level with regional population estimates from the Office of National Statistics [16]. We found THIN to be highly representative in terms of age and sex structure at the regional level. Population pyramids showing the representativeness of THIN by age and sex on a regional basis are presented as supplementary material (Additional file 1). Since both GLF and THIN are regionally representative, the prevalence of smoking was compared between these two data sources directly, without standardisation for age or sex. To ensure that this assumption was appropriate, we also calculated age and sex-standardised estimates, calculated through indirect standardisation by applying age- and sex-specific smoking rates from the corresponding GLF data to the THIN population. These are extremely similar to the unstandardised estimates and are shown in additional file 2.

Annual estimates of smoking prevalence in the different regions based on THIN were compared graphically with point estimates, and confidence intervals around those estimates, from the GLF, to assess whether regional estimates of prevalence in THIN are similar to those from the GLF. Confidence intervals were not drawn for THIN as its much larger sample size results in precise estimates and very narrow confidence intervals which are not easily graphically distinguishable from the point estimates. These confidence intervals are shown in additional file 3.

The data used in this study form part of the recently-developed Nottingham Tobacco Control Database, a compilation of sources of smoking-related information at a national and regional level. All analysis was carried out in Stata Version 11.0 (Stata Corp, College Station, TX) and the analysis of THIN data for this study was approved by the Derbyshire Research Ethics Committee.

## Results

Table 1 below shows the sample sizes of the GLF and THIN by region in 2000 and 2008, demonstrating the much-reduced sample sizes in the regional data. The regional sample sizes in THIN remain extremely large, with over 100,000 people in each region in 2008. The regional GLF sample sizes are much smaller, ranging from 642 in the North East region to 2,082 in the South East in 2008.

**Table 1:** Sample sizes of General Lifestyle Survey and THIN, 2000 and 2008

	GLF 2000	THIN 2000	GLF 2008	THIN 2008
North East	686	99,054	642	109,322

North West	1,701	286,949	1,691	316,195

Yorks And Humber	1,253	136,982	1,370	144,646

East Midlands	997	130,418	1,222	140,882

West Midlands	1,300	285,633	1,354	317,585

East Of England	1,348	222,265	1,497	243,355

London	1,504	293,421	1,207	338,859

South East	2,062	525,110	2,082	704,886

South West	1,303	290,763	1,420	350,742

Wales	724	141,372	830	210,585

Scotland	1,211	198,045	1,304	250,109

**Total**	**14,089**	**2,703,864**	**14,619**	**3,245,031**

**Figure 1:**
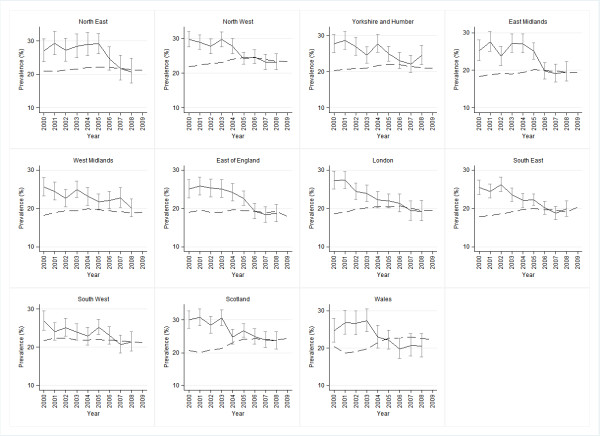
**Smoking prevalence by region from THIN and GLF (2000-2008)**. Solid line: GLF Dashed line: THIN

Figure 1 shows the comparison of THIN and GLF smoking prevalence data by region from 2000 to 2008. The GLF data show the general decreasing trend in smoking prevalence in recent years in all regions. In most regions, prevalence estimates from THIN converged with those from the GLF over the years of the study, with good agreement between the data sources, and THIN estimates falling within the confidence intervals of the GLF, from 2006 onwards.

In three regions this convergence is not observed. The data from the West Midlands did not converge during the study period, although the discrepancy between the two datasets fell, and the THIN estimate was within the confidence interval for the GLF estimate in the final year. The Yorkshire and Humber data converged during the study period but the 2008 THIN estimate did not fall within the GLF confidence interval. The data from Wales converged from 2001 to 2005, at which point prevalence as measured by both data sources was approximately 22%. In subsequent years the values moved apart again; however, the THIN estimates were within the confidence interval for the GLF in the final two years of the study.

## Discussion

To our knowledge, this is the first study to validate primary care smoking data at the regional level. These results show that estimates of regional smoking prevalence from THIN are highly comparable to the corresponding estimates from the current main source of such data. In most regions, smoking prevalence based on THIN data was similar to that found by the GLF from 2006 onwards. Primary care data could therefore be used to help target tobacco control initiatives at the areas with the highest smoking prevalence and to monitor prevalence across regions.

The main limitation of our study is that we were unable to compare THIN data with the corresponding data for all of the UK's regions. The GLF covers Great Britain only, and therefore we could not validate prevalence data for Northern Ireland. However, our results were generally consistent across all regions that were included, and it is likely that THIN smoking prevalence estimates for Northern Ireland are similarly accurate. Further to this, we were unable to explore the comparability of THIN and GLF prevalence estimates beyond 2008. Estimates from these two data sources were similar in the final three years of the study only; further research will be required in the future to ascertain whether this agreement is maintained in subsequent years.

A further limitation of our study is that the GLF and THIN may underestimate smoking prevalence, as both GLF respondents and general practice patients do not have their smoking status biochemically validated. However, the high costs associated with such validation mean that it is extremely difficult to obtain it for such large samples. In addition, because there is considerable variation in the completeness of recording between UK general practices, these results are not necessarily generalisable across all practices.

A final limitation of this study is that the significantly diminished sample sizes of the GLF at the regional level mean that there may be significant error in its estimates. However, during the study period the GLF was the largest survey providing regional prevalence data for Great Britain, and we believe that this is therefore the most appropriate comparator.

Despite the diminished sample size of the THIN data at the regional level, the results of this study are broadly consistent with those of the previous validation study of these data carried out at the national level. As at the national level, prevalence estimates based on THIN from most regions were found to be similar to those based on the GLF from 2006 [10].

The convergence in prevalence estimates from THIN and the GLF is almost certainly a result of the voluntary, pay-for-performance general practice contract introduced in 2004 [17]. The contract requires GPs to record their patients' smoking status at least every 27 months (every 15 months for patients with specified chronic diseases) and has been taken up by almost all GPs [18].

Convergence between the two datasets by 2006 was not observed in all regions; there was greater discrepancy between the data sources for the West Midlands, Yorkshire and the Humber and Wales. Regional GLF data are based on small sample sizes, with resultant higher sampling error, as demonstrated by the wide confidence intervals, and any discrepancy between THIN and GLF estimates may reflect uncertainty associated with the GLF data rather than inadequacy of estimates from THIN. Further to this, while we were able to confirm that THIN is representative regionally in terms of age and sex, we have not assessed representativeness in terms of other factors such as social class. This may also account for some of the discrepancy in the three aforementioned regions. That even in these regions, the THIN estimates in two of the final three years (Yorkshire and Humber), the final year (West Midlands) and the final two years (Wales) of the study were within the confidence intervals of the GLF estimates demonstrates that estimates from GLF and THIN for these regions may indeed be comparable. The discrepancy in the final year of data for Yorkshire and Humber may be due to young adults being underrepresented in the THIN population of this region in the final year of the study (as shown in additional file 1).

There are several advantages to using THIN prevalence data compared with the national survey data. THIN data are routinely collected, are released 3-4 times per year, and have a lag of only 3-8 months before data become available [10]. A further advantage is its size; the standard error of THIN's smoking prevalence estimates is significantly smaller than those of the GLF at a national level [10]. At a regional level, GLF estimates are prone to more error due to much reduced sample sizes and confidence intervals are so wide, as demonstrated in Figure 1, that changes from year to year will be difficult to detect; therefore the large sample size in THIN is extremely valuable. Further to this, THIN provides monthly data, which is particularly useful in the evaluation of short term impacts of tobacco control initiatives.

Based on the THIN data, it was found that in 2008 Scotland (24%), the North West (23.5%), and Northern Ireland (23.5%) had the highest smoking prevalence in the UK. The East of England (19%), the West Midlands (19%) and South East England (19%) had the lowest prevalence. There remains substantial variation in smoking prevalence between the regions, with higher prevalence often being observed in regions with the lowest per capita disposable income [19]. Smoking is an important contributor to health inequalities [20,21]. Therefore, reducing regional differences in smoking prevalence will contribute to alleviating health inequalities in the UK. This study indicates that THIN may be a useful source of data for monitoring these regional differences.

To our knowledge, the current study and that by Szatkowski et al. are the first to explore the possibility of using primary care data to monitor smoking prevalence [2]; our results indicate that primary care data are a potentially valuable source of such information. Previous research suggests that surveys which monitor smoking prevalence in EU Member States often have small sample sizes and are irregularly carried out [22]. This suggests that the way in which smoking prevalence is monitored internationally has similar limitations to the way it is currently monitored in Britain. Future research exploring the possibility of using primary care data to monitor smoking prevalence in countries other than Britain may therefore be warranted.

## Conclusions

It is important to monitor regional patterns of smoking prevalence to ensure tobacco control measures in the UK are targeted at the areas most in need and help to reduce the health inequality caused by smoking. THIN data on smoking prevalence at the regional level are comparable with the main source of UK data on this measure, and could therefore be used to monitor longitudinal regional trends in smoking prevalence.

## Competing interests

The authors declare that they have no competing interests.

## Authors' contributions

TL participated in the design of the study and drafted the manuscript. LS extracted the study data from the THIN database and revised the manuscript for intellectual content. SW carried out the analysis of the data and helped to draft the manuscript. SL participated in the design of the study and revised the manuscript for intellectual content. All authors read and approved the final manuscript.

## Pre-publication history

The pre-publication history for this paper can be accessed here:

http://www.biomedcentral.com/1471-2458/11/773/prepub

## Supplementary Material

Additional file 1 **Representativeness of THIN by region in 2000 and 2008**. Population pyramids showing the representativeness of THIN by age and sex on a regional basis (based on population estimates from ONS) Click here for file

Additional file 2 **Smoking prevalence by region from THIN and GLF (2000-2008) using standardised GLF data**. Figure 1 from main manuscript re-drawn using age and sex-standardised GLF data Click here for file

Additional file 3 **Smoking prevalence by region from THIN and GLF (2000-2008) with 95% confidence intervals for THIN**. Figure 1 from main manuscript re-drawn showing confidence intervals for THIN estimates. Click here for file
