# Supplementary figures and images for: Can primary care data be used to monitor regional smoking prevalence? An analysis of The Health Improvement Network primary care data
Source: BMC Public Health. 2011 Oct 7;11:773. doi: 10.1186/1471-2458-11-773 (PMC3198710; doi:10.1186/1471-2458-11-773)

**Representativeness of THIN by region in 2000 and 2008**

2000


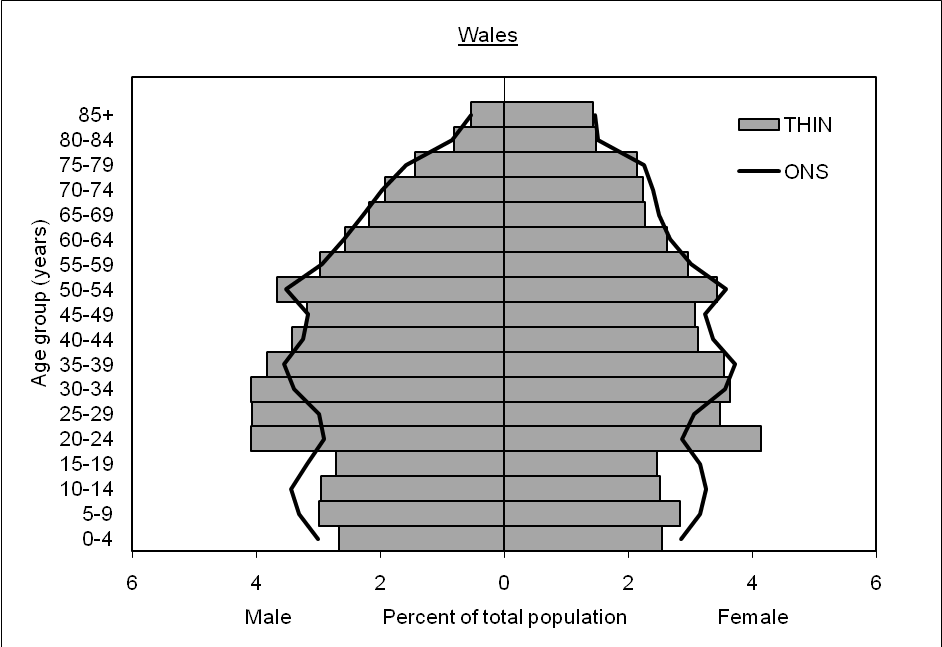

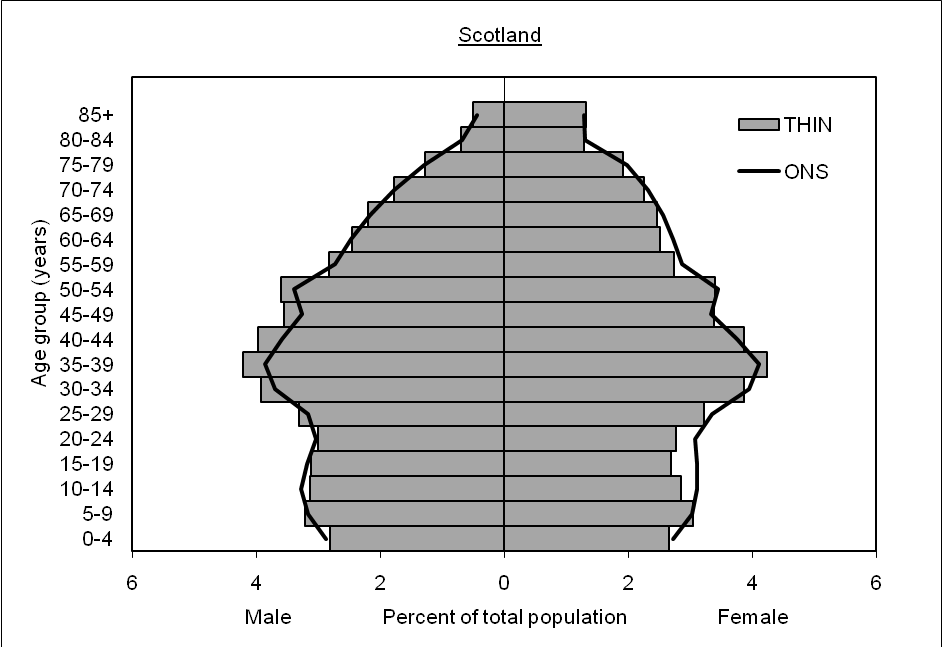

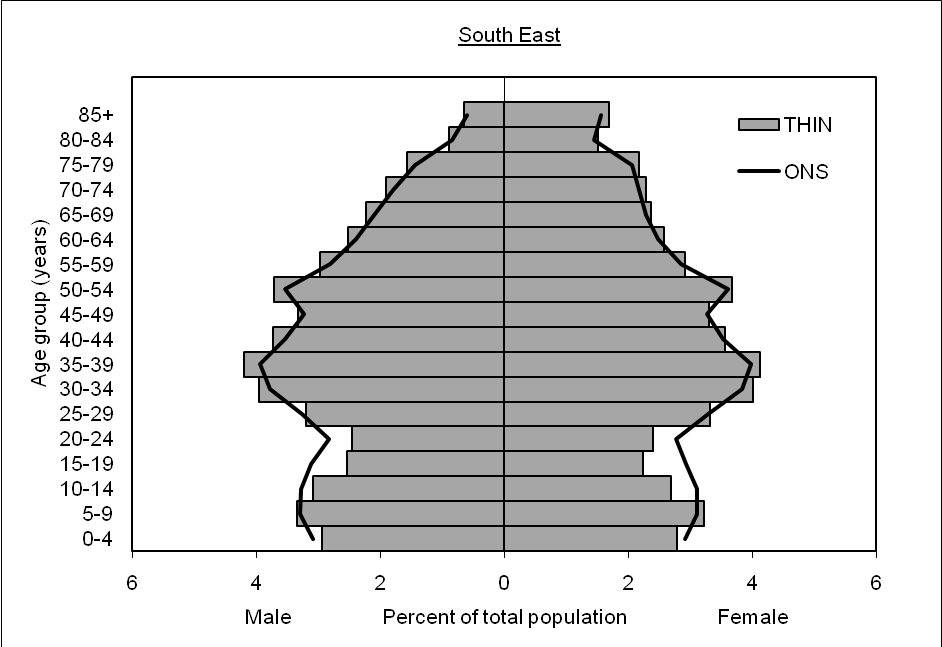


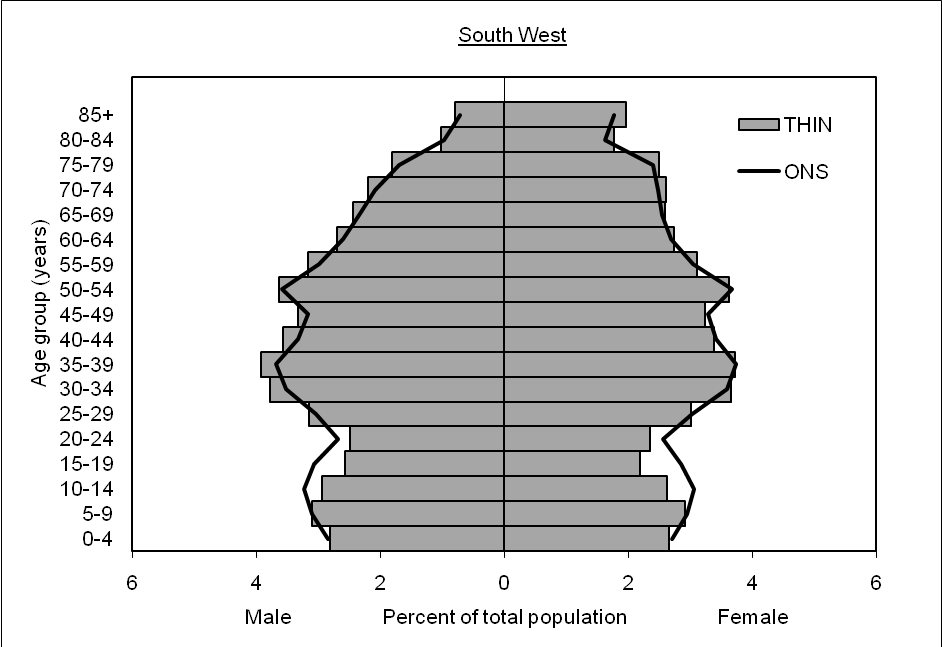

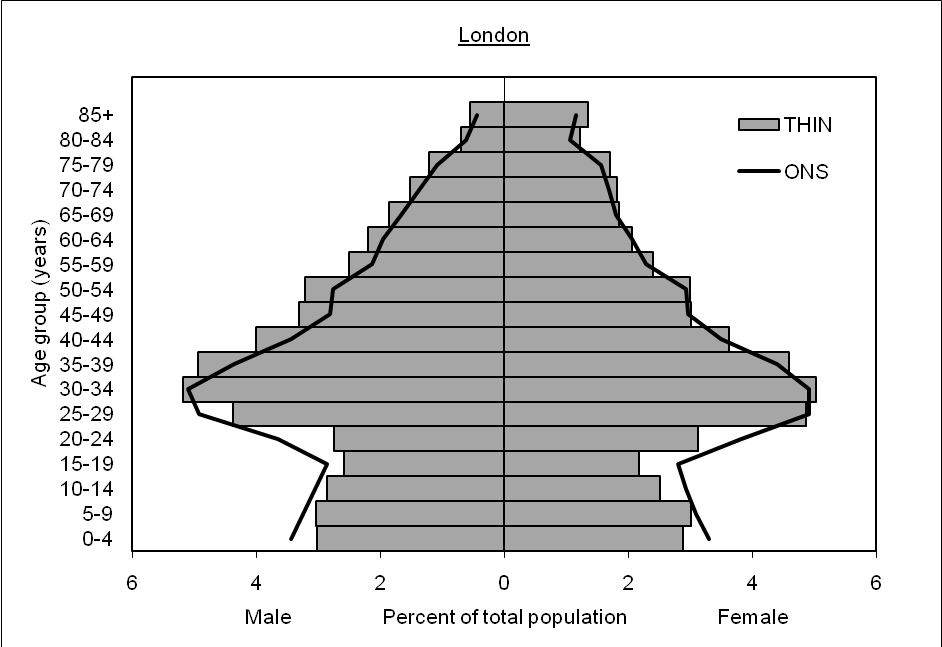


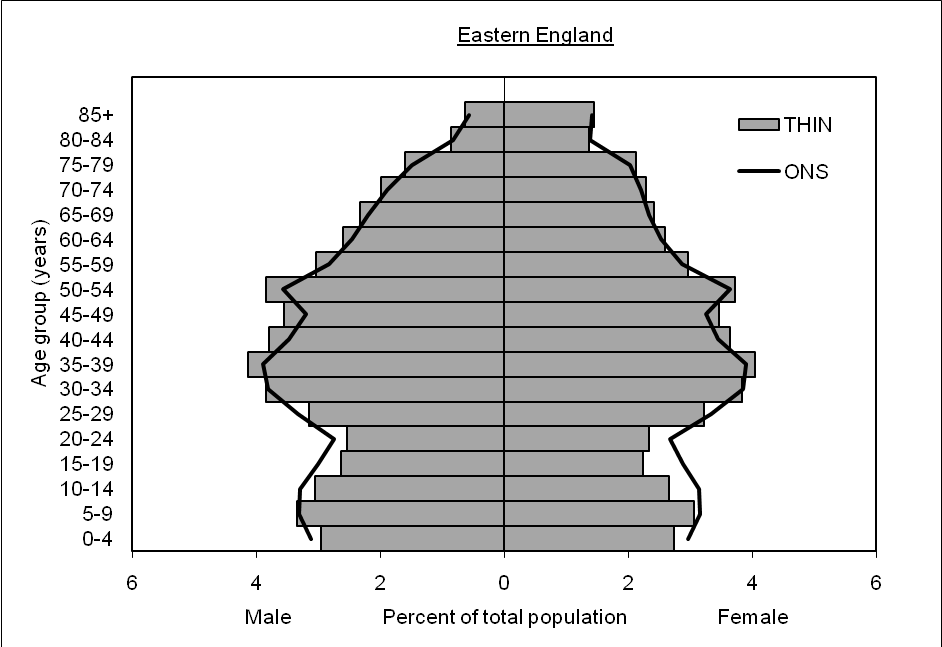

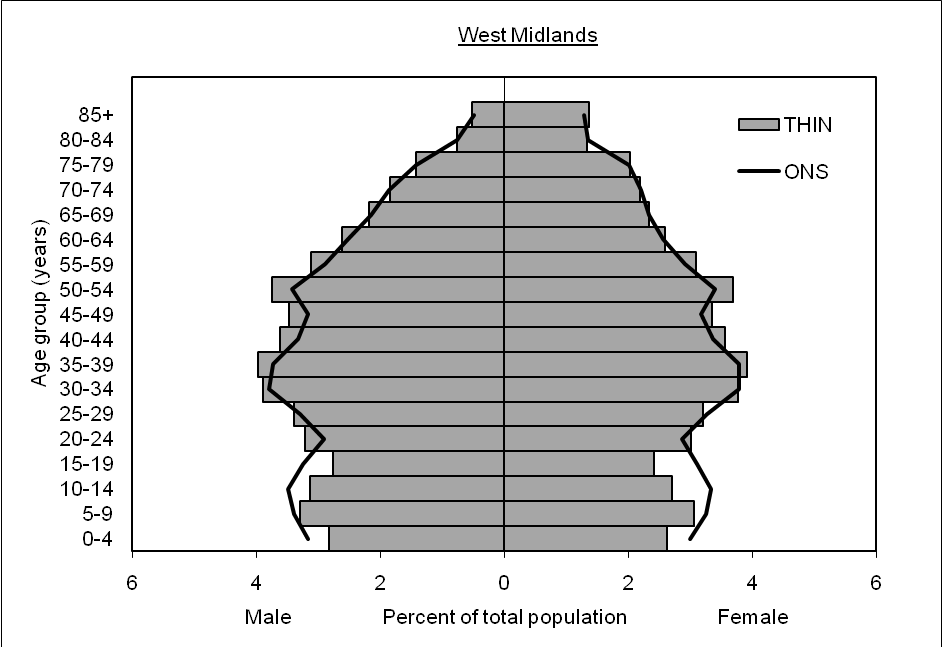


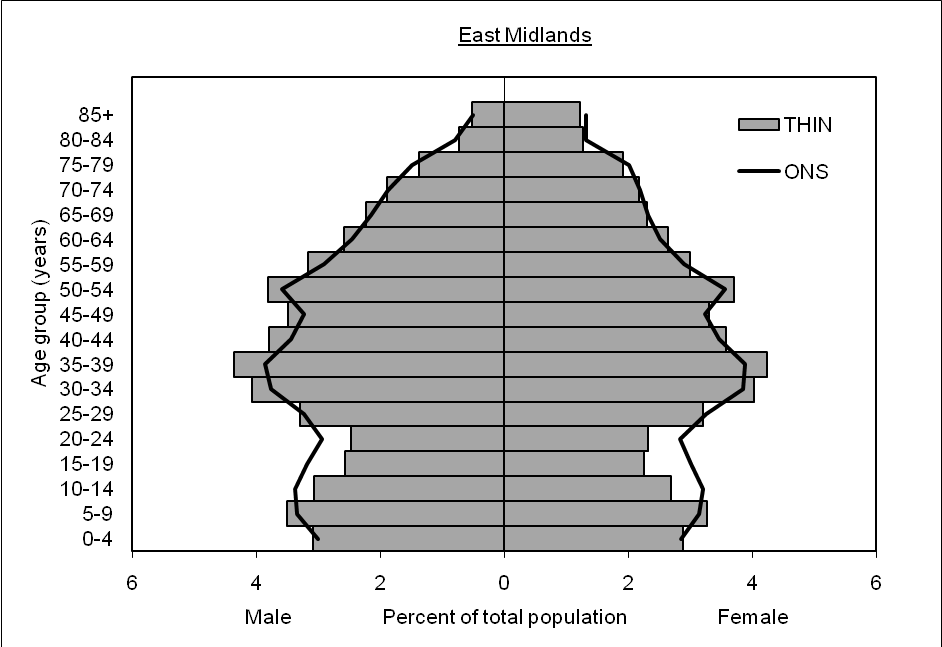

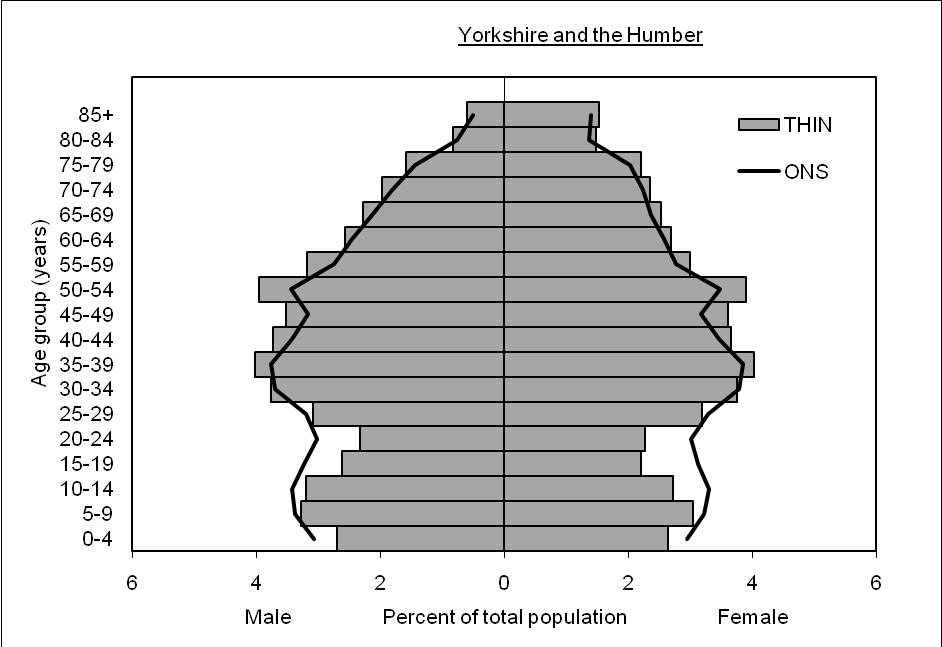


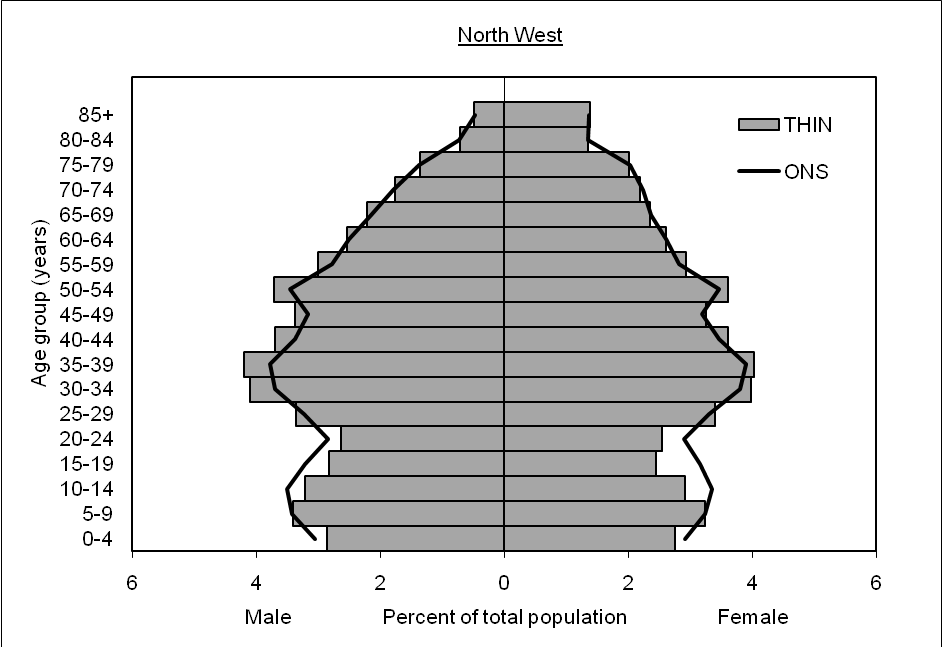

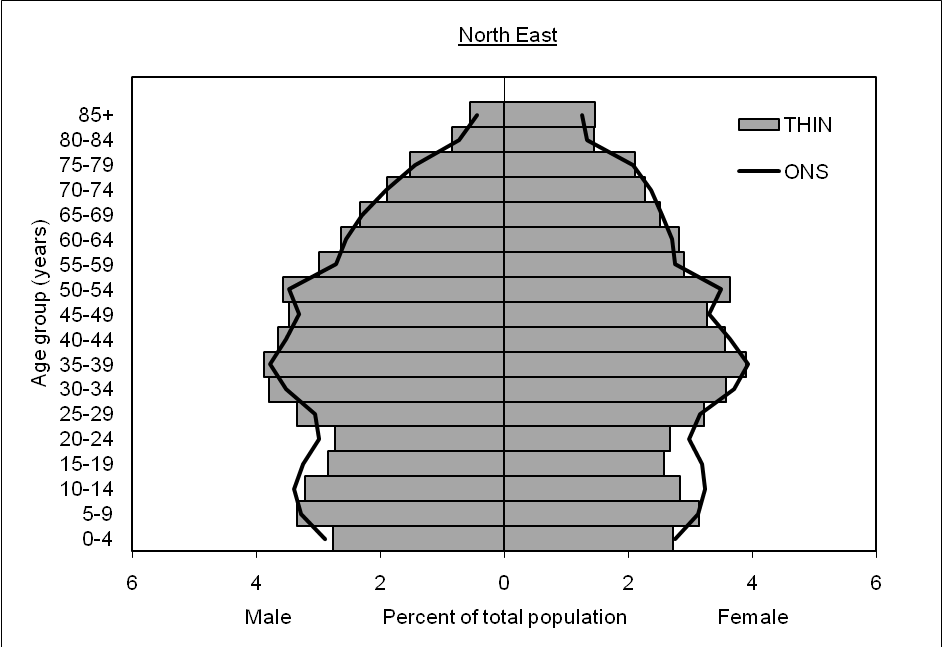


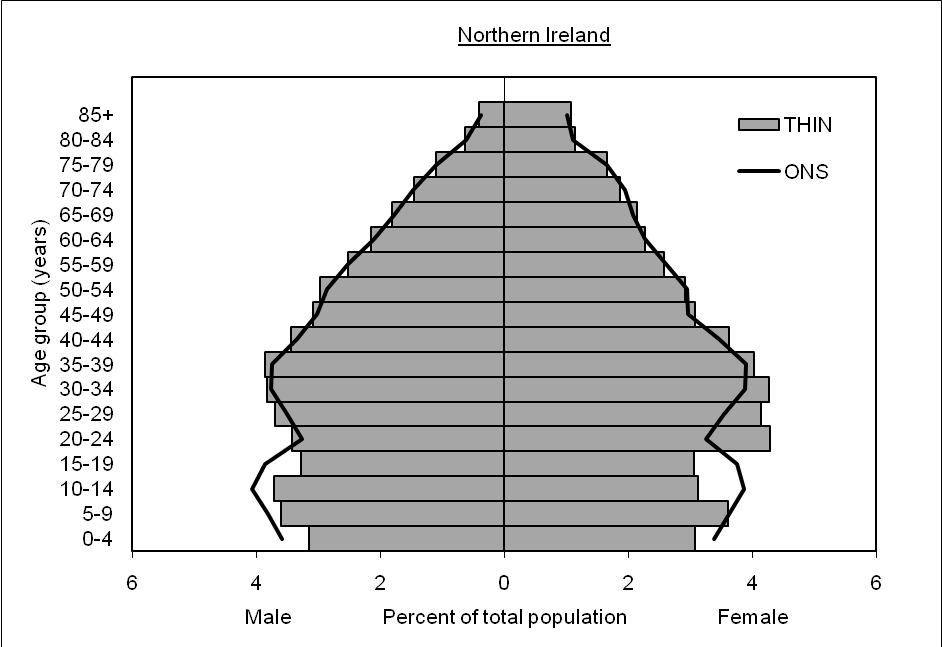


2008


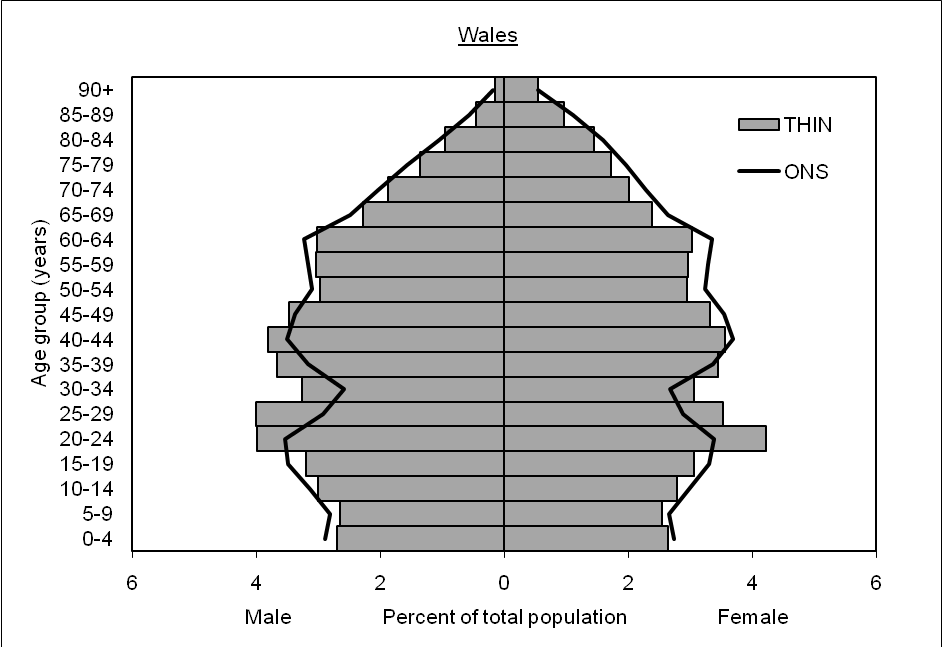


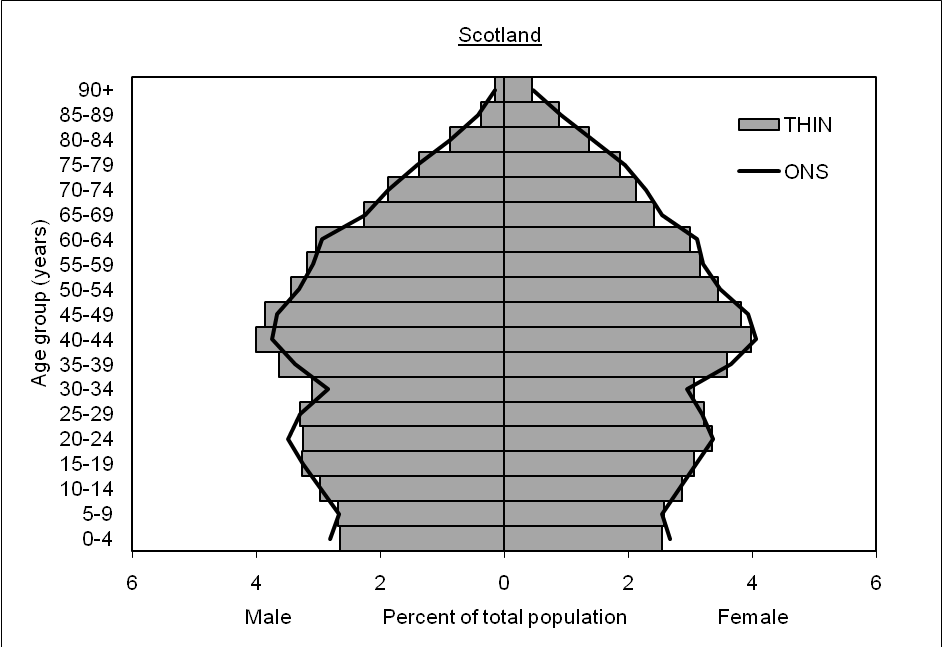


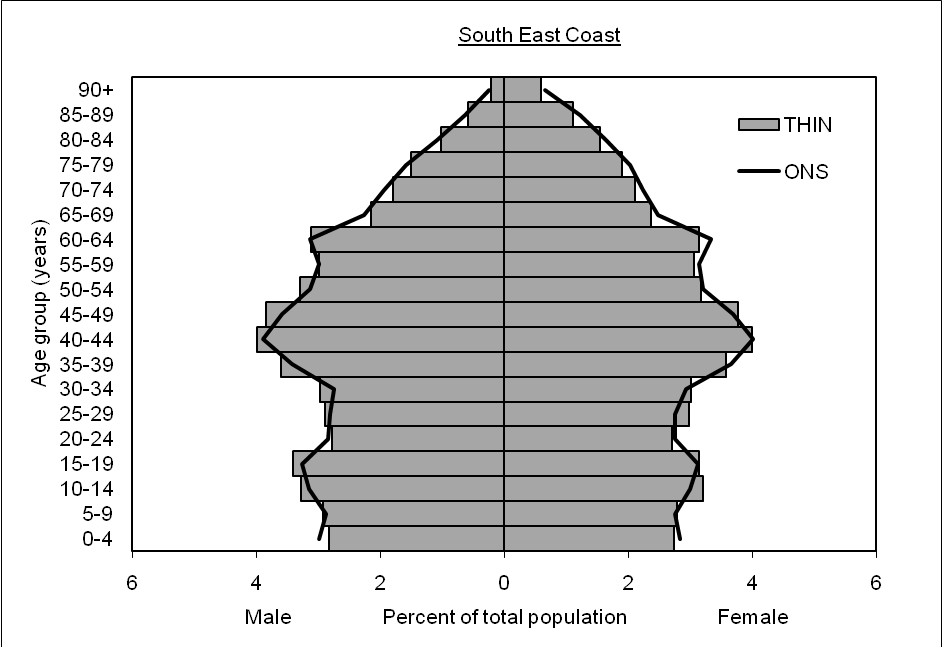


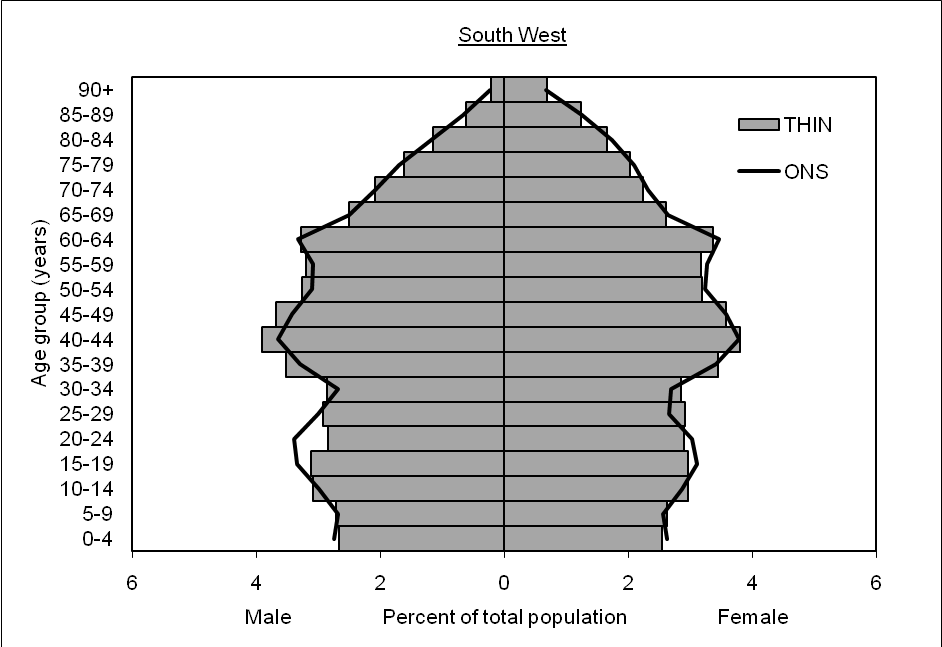

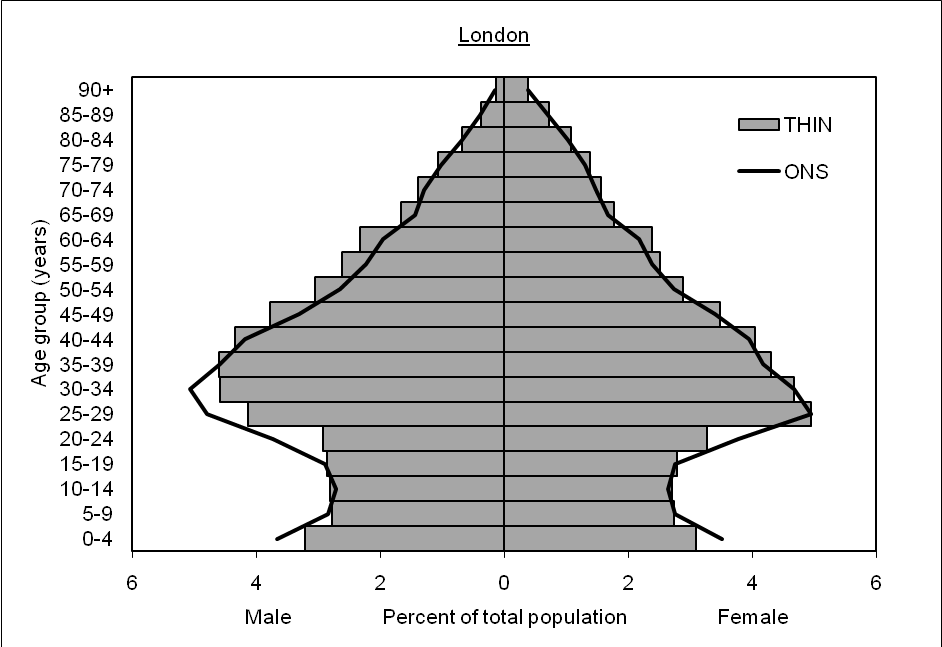


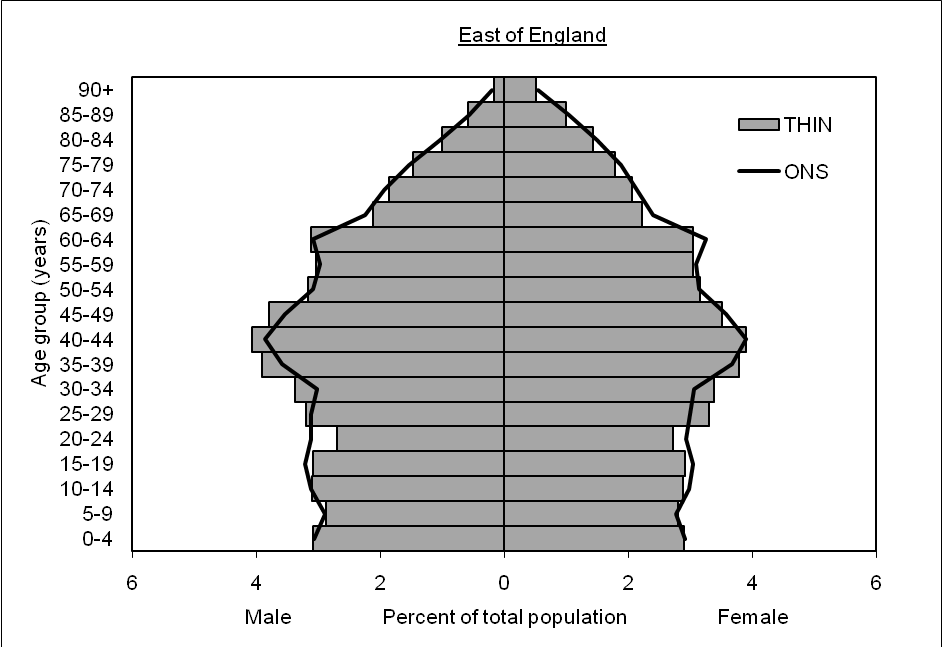

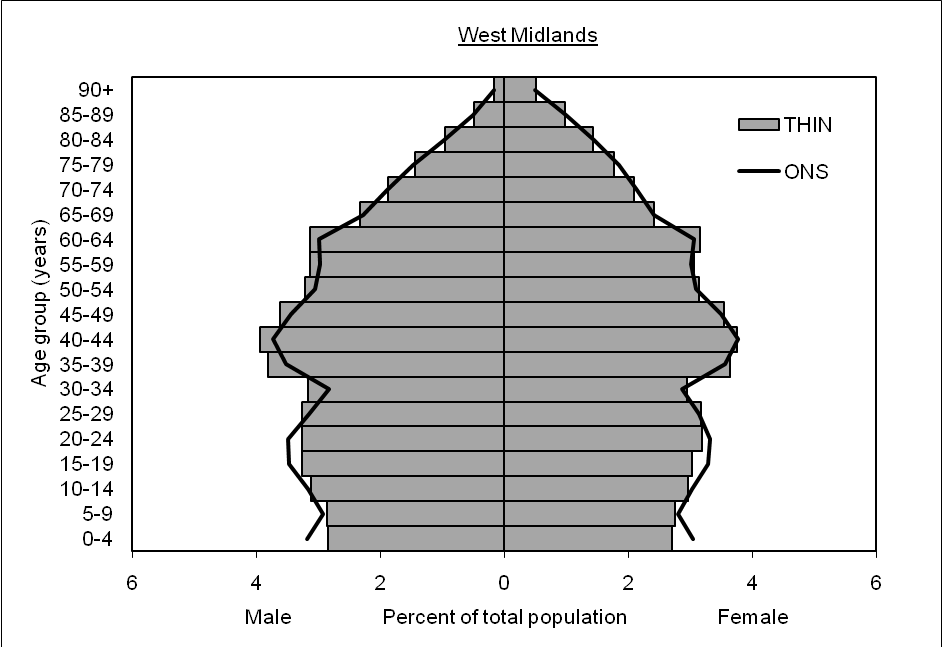


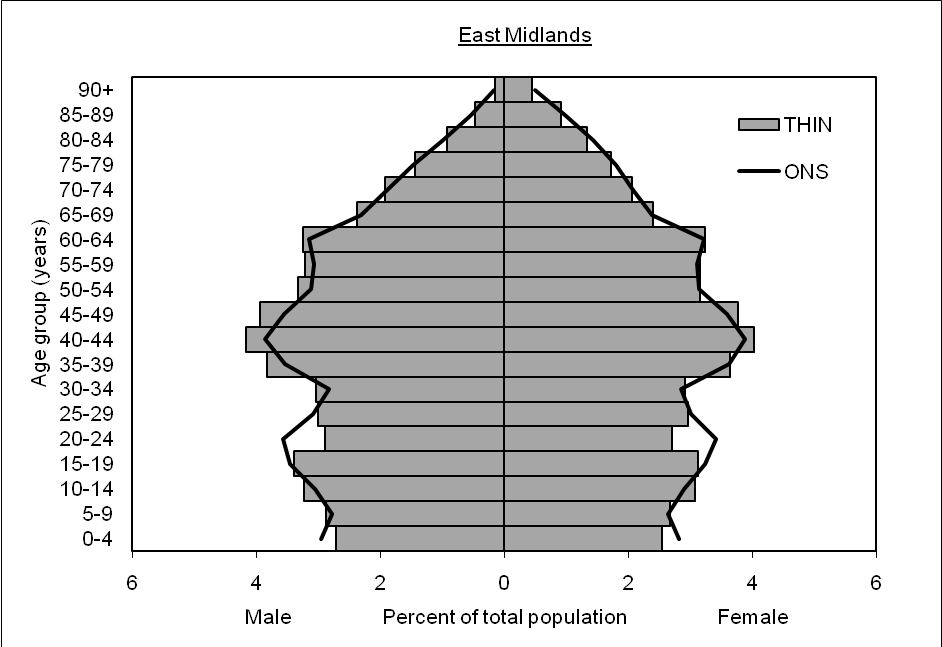

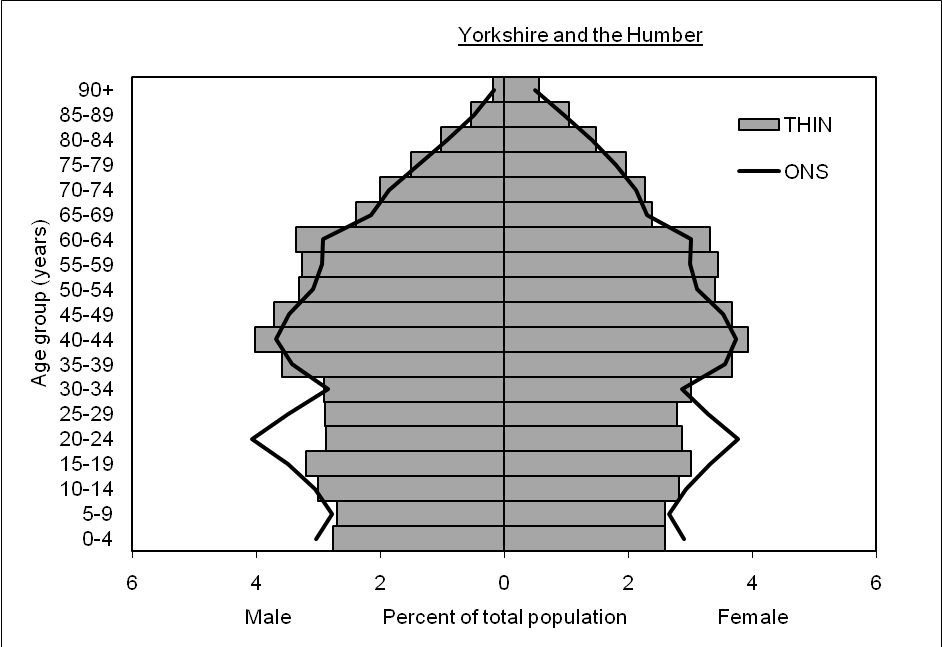


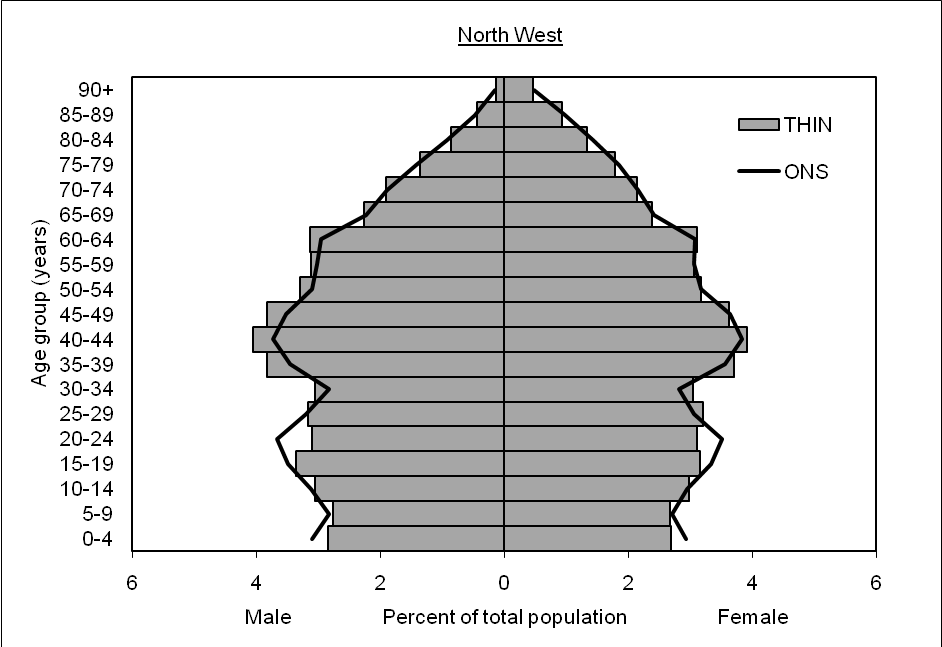

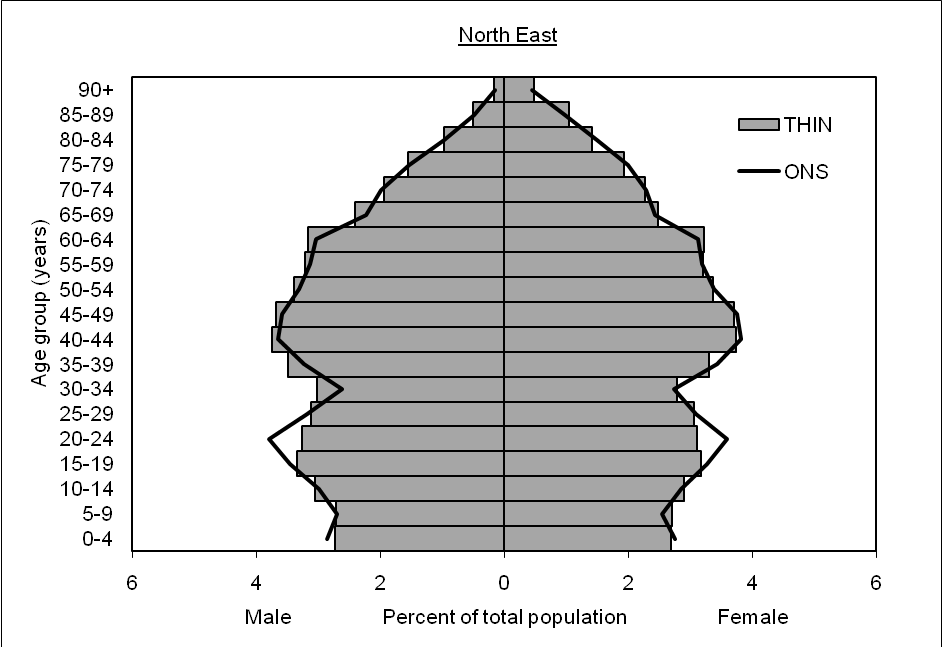


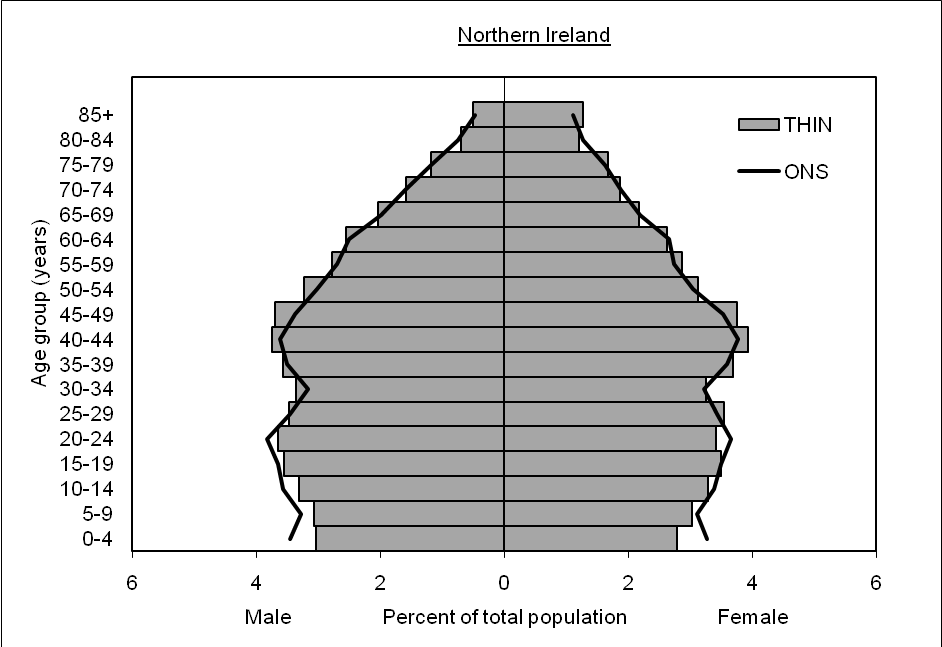

Supplement: Additional file 1 — Representativeness of THIN by region in 2000 and 2008. Population pyramids showing the representativeness of THIN by age and sex on a regional basis (based on population estimates from ONS) [file 1471-2458-11-773-S1.DOC]
