# Supplementary material for: Can primary care data be used to monitor regional smoking prevalence? An analysis of The Health Improvement Network primary care data
Source: BMC Public Health. 2011 Oct 7;11:773. doi: 10.1186/1471-2458-11-773 (PMC3198710; doi:10.1186/1471-2458-11-773)

**Supplementary file 2. Smoking prevalence by region from THIN and GLF (2000-2008) using standardised GLF data**


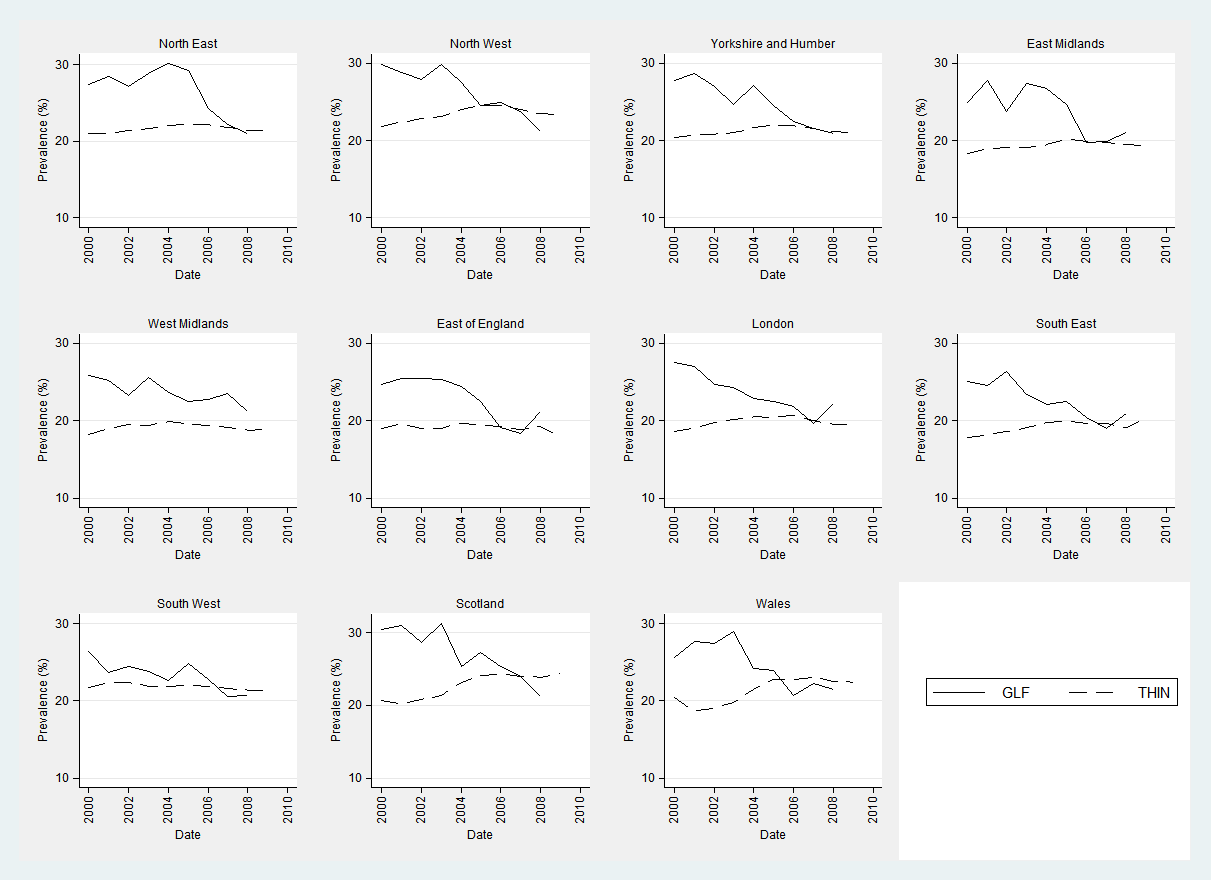

Supplement: Additional file 2 — Smoking prevalence by region from THIN and GLF (2000-2008) using standardised GLF data. Figure 1 from main manuscript re-drawn using age and sex-standardised GLF data [file 1471-2458-11-773-S2.DOC]
