# Supplementary material for: Can primary care data be used to monitor regional smoking prevalence? An analysis of The Health Improvement Network primary care data
Source: BMC Public Health. 2011 Oct 7;11:773. doi: 10.1186/1471-2458-11-773 (PMC3198710; doi:10.1186/1471-2458-11-773)

**Supplementary file 3. Smoking prevalence by region from THIN and GLF (2000-2008) with 95% confidence intervals for THIN**
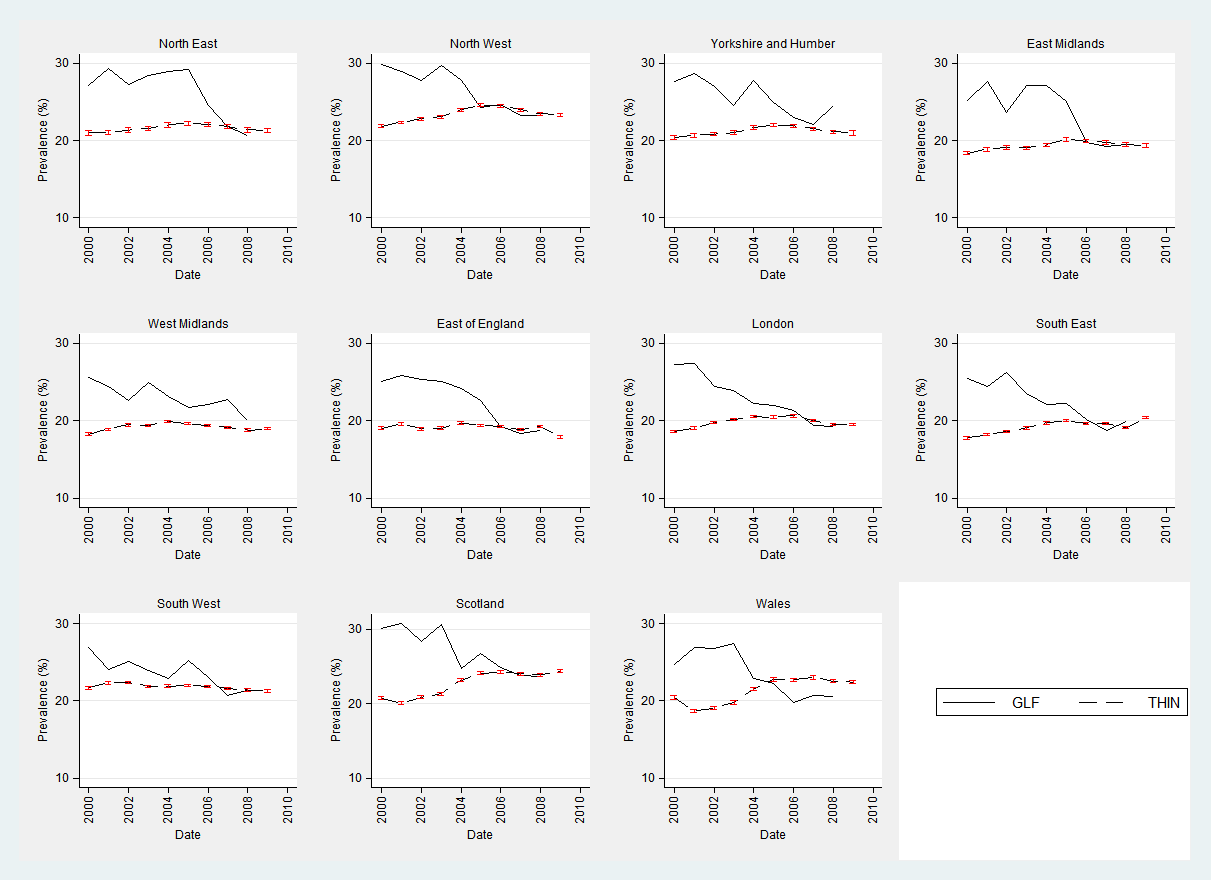

Supplement: Additional file 3 — Smoking prevalence by region from THIN and GLF (2000-2008) with 95% confidence intervals for THIN. Figure 1 from main manuscript re-drawn showing confidence intervals for THIN estimates. [file 1471-2458-11-773-S3.DOC]
